# The Glucagon-Like Peptide-1 (GLP-1) Receptor Agonist Liraglutide Regulates Sirtuin-1-Mediated Neutrophil Extracellular Traps to Improve Diabetes-Induced Bone Metabolism Imbalance

**DOI:** 10.5812/ijpr-148139

**Published:** 2024-11-13

**Authors:** Shuai Zhong, Liangzhi Huang, Tingting Lin, Yanyan Li, Bin Deng, Dezhi Kong, Zhanlin Liao, Zugui Huang

**Affiliations:** 1 Department of Endocrinology, The Affiliated Nanping First Hospital, Fujian Medical University, Nanping, Fujian, China

**Keywords:** Diabetes Mellitus, Bone Metabolism, Liraglutide, Neutrophil Extracellular Traps, SIRT1

## Abstract

**Background:**

Diabetes mellitus (DM) is a chronic metabolic disorder that disrupts normal bone remodeling.

**Objectives:**

This study aimed to investigate how the glucagon-like peptide-1 (GLP-1) receptor agonist liraglutide (LIR) addresses bone metabolism imbalances induced by type-II diabetes.

**Methods:**

Type-II diabetic rat models were established through a single intraperitoneal injection of streptozotocin (STZ). Blood glucose levels were measured using a blood glucose meter, and insulin levels were assessed using an assay kit. Bone formation markers [alkaline phosphatase (ALP), osteocalcin (OCN), and procollagen I N-terminal propeptide (PINP)] and bone resorption markers [tartrate-resistant acid phosphatase (TRACP) and CTX-1] were monitored using assay kits. Bone marrow mesenchymal stem cells (BMSCs) were cultured in vitro under high-fat and high-glucose (HFHS) conditions to mimic diabetic bone metabolism dysregulation. Neutrophil extracellular traps (NETs) formation was examined through immunofluorescent staining and Western blot analysis.

**Results:**

Liraglutide was found to reduce STZ-induced NETs formation, as indicated by decreased expression of cit-H3 by 36.90% - 53.57%, myeloperoxidase (MPO) by 55.81% - 65.12%, NE by 53.95% - 65.17%, and PAD4 by 46.81% - 63.83%, alongside increased Sirtuin-1 (SIRT1) expression in femur tissue (70.71% - 91.19%). In vitro, LIR enhanced osteogenesis and inhibited apoptosis, effects that were partially reversed by SIRT1 knockdown. Additionally, SIRT1 knockdown partially restored LIR-induced reductions in oxidative stress, inflammation, and NETs formation.

**Conclusions:**

LIR mitigates diabetes-induced bone metabolism imbalance by inhibiting NETs formation through SIRT1 mediation.

## 1. Background

Diabetes mellitus (DM) is a group of chronic metabolic disorders characterized by elevated blood glucose levels (1). Affecting over 440 million people globally—approximately 8.8% of the adult population—DM places a heavy burden on healthcare resources (2). This disruption in glucose metabolism leads to complications in most organ systems of diabetic patients. In hyperglycemia, the imbalance between bone resorption and formation during remodeling results in increased bone resorption (3). Consequently, DM prolongs resorptive activity, increases detachment, accelerates bone loss, and impairs new bone formation (4). Diabetes-related bone disease, characterized by elevated fracture risk and reduced bone healing, presents a major challenge to healthcare providers (5).

The incretin hormone glucagon-like peptide-1 (GLP-1) is released by L-cells in the intestinal wall, where it enhances insulin secretion and lowers blood glucose levels (6). However, GLP-1 is naturally broken down in the bloodstream within minutes (7). To extend its physiological effects in vivo, various GLP-1 receptor agonists (GLP-1RAs) have been developed. Liraglutide (LIR), a leading GLP-1RA, is now a preferred therapeutic agent for individuals with type 2 diabetes mellitus (T2DM) because it promotes weight loss, improves insulin resistance, and lowers blood glucose in T2DM patients and in animal models (8).

Sirtuin-1 (SIRT1), an NAD-dependent enzyme involved in deacetylation, is commonly linked to longevity genes (9). Recent animal studies suggest that SIRT1 could be a valuable pharmacological target for treating osteoporosis and other bone conditions. Preclinical findings show that mice treated with SIRT1 activators have increased resistance to osteoporosis in models of aging and post-menopause (10). Nicotinamide mononucleotide has been shown to promote bone formation and reduce adipogenesis by regulating mesenchymal stromal cells through the SIRT1 pathway in aged bone marrow (11). In our study, we investigated SIRT1 expression in femur tissue from diabetic rats, finding that LIR treatment significantly increased the STZ-reduced SIRT1 levels.

Neutrophil extracellular traps (NETs), which consist of histones and antimicrobial proteins from neutrophils, are released by activated neutrophils into the extracellular space to combat pathogens (12). Produced by activated neutrophils, NETs also act as autoantigens and play a significant role in the progression of bone and joint disorders by influencing inflammatory factor expression (13). However, excessive accumulation and activation of neutrophils are associated with immune-related tissue damage in various disease states (13). Research has shown that in mice with imiquimod (IMQ)-induced lupus-like disease treated with methylprednisolone (mPSL) pulses, neutrophil infiltration led to the formation of NETs in the tissue, resulting in cartilage ischemia in the femoral head (14). In periodontitis, NETs are pivotal in initiating pathogenic inflammation as neutrophils infiltrate the gingival mucosa, release NETs, and thereby contribute to mucosal inflammation and bone destruction (15).

## 2. Objectives

This study aimed to investigate how the GLP-1 receptor agonist LIR addresses bone metabolism imbalances induced by type-II diabetes.

## 3. Methods

### 3.1. Diabetic Rat Model Construction 

Male Sprague-Dawley rats, aged six weeks and weighing 200 - 220 gr, were obtained from the Cavens Experimental Animal Center in Changzhou, China. The animals were housed in a controlled environment at a temperature of 23°C with a 12-hour natural light/dark cycle and provided with water and a standard diet. The rats were divided into three groups: The control group (N = 5), the streptozotocin (STZ) group (N = 5), and the STZ+LIR group (N = 5). Throughout the study, the control group received a standard rodent diet, while the other two groups were given a high-fat diet (D12492, Research Diets, New Brunswick, NJ, USA) consisting of 60% calories from fat, 20% from carbohydrates, and 20% from protein (16) for a period of 8 weeks.

After 8 weeks on the high-fat diet, the rats in the STZ and STZ+LIR groups received a 30 mg/kg (17) intraperitoneal injection of STZ (HY-13753, MedChemExpress, Shanghai, China), while rats in the control group were administered an equivalent dose of citrate buffer. Fasting blood glucose (FBG) levels were measured one week post-STZ injection. Rats with FBG levels over 16.7 mmol/L were classified as diabetic (18). One week after the STZ injection, rats in the STZ+LIR group began receiving a daily subcutaneous injection of 0.2 mg/kg LIR (HY-P0014, MedChemExpress, Shanghai, China) (19). The control and STZ groups received equivalent doses of normal saline. Body weight was recorded regularly. 

All rats were maintained under these conditions for 12 weeks and then euthanized using the CO₂ method. Blood samples were collected and stored at -80°C. The left femurs of the rats were harvested, rinsed with phosphate-buffered saline (PBS), and preserved at -80°C. The Experimental Animal Ethics Committee of Fujian Anburui Biotechnology (Approval No: IACUC-FJABR2023026020) ensured that all animal handling and experimental procedures adhered to the guidelines of the World Medical Association’s Declaration of Helsinki.

### 3.2. Identification of Blood Glucose, Insulin, and Bone Metabolism Markers 

Blood samples were centrifuged at 2,000 g for 10 minutes, and the serum supernatant was then frozen at -80°C for later analysis. Serum insulin levels were measured using a rat insulin ELISA kit (D731159-0096, Sangon, Shanghai, China), following the manufacturer's instructions. Blood glucose levels were determined using a Roche blood glucose meter (Roche, Basel, Switzerland). Serum levels of alkaline phosphatase (ALP, D799817-0500, Sangon, Shanghai, China), osteocalcin (OCN, D731045-0096, Sangon, Shanghai, China), procollagen I N-terminal propeptide (PINP, D731148-0096, Sangon, Shanghai, China), tartrate-resistant acid phosphatase (TRACP, E-EL-R0939, Elabscience, Wuhan, China), and c-terminal telopeptide of type 1 collagen (CTX-1, D731151-0096, Sangon, Shanghai, China) were measured using ELISA kits, following each manufacturer's instructions.

### 3.3. Immunofluorescence Staining

After sacrifice, the rat femurs were dissected and adherent muscle tissue was removed. The bones were fixed in 10% formalin at 4°C overnight. Following washing in PBS, the femurs were decalcified in 0.5 M EDTA (pH 7.4) at 4°C with continuous agitation for 3 days. The samples were then dehydrated in a solution containing 20% sucrose and 2% polyvinylpyrrolidone for 24 hours. Subsequently, the tissues were embedded in OCT, and longitudinal sections of 20 μm thickness were prepared for staining. 

The sections were incubated overnight at 4°C with primary antibodies against citrullinated histone H3 (cit-H3, 1:1500, 97272S, CST, MA, USA) and then treated with Cy5-conjugated secondary antibodies (1:200, ab6565, Abcam, Shanghai, China) for 1 hour in the dark at room temperature. DAPI (1 μg/mL, C0065, Solarbio, Beijing, China) was used to counterstain the nuclei for 5 minutes. Observations were conducted using a confocal microscope (Olympus Confocal FV1000 Microscope).

### 3.4. Western Blot Analysis 

Tissue or cell samples were thoroughly homogenized in a glass blender and then mixed with 400 μL of RIPA lysis buffer at 4°C for 30 minutes. The lysates were then centrifuged at 13,000 rpm for 10 minutes at 4°C. Protein supernatants were collected for quantification using an enhanced BCA Assay Kit (P0011, Beyotime, Shanghai, China). Equal amounts of protein (∼30 μg per sample) were subjected to SDS-PAGE electrophoresis. After transfer to PVDF membranes, the blots were blocked with a 5% (w/v) non-fat dried milk solution containing 5% BSA at 37°C for 2 hours. Membranes were then incubated overnight at 4°C with primary antibodies, including cit-H3 (1:1000, 97272S, CST, MA, USA), myeloperoxidase (MPO, 1:2000, 22225-1-AP, Proteintech, Wuhan, China), neutrophil elastase (NE, 1:1000, ab310335, Abcam, Shanghai, China), peptidyl arginine deiminase 4 (PAD4, 1:1000, ab214810, Abcam, Shanghai, China), NAD-Dependent Protein Deacylase SIRT1 (1:1500, ab110304, Abcam, Shanghai, China), and GAPDH (1:2500, ab9485, Abcam, Shanghai, China). Afterward, the blots were incubated with HRP-conjugated secondary antibodies (1:10000) at room temperature for 4 hours. Following three 10-minute washes in TBST, signals were visualized using ECL reagents for 2 minutes (Pierce, MA, USA) and imaged with the FluorChem system (BioRad Lab, CA, USA).

### 3.5. Cell Culture and Treatment 

Rat bone marrow stromal cells (BMSCs) were obtained as follows: Four-week-old SD rats were sacrificed and soaked in 75% alcohol for 10 minutes. Their femurs and tibias were removed, rinsed with PBS under sterile conditions, and the bone marrow cavities flushed to obtain a cell suspension. Cells were cultured in a 60 mm dish at 37°C in a humidified environment with 5% CO₂. Dulbecco’s Modified Eagle Medium (DMEM, 12491015, Gibco, MA, USA) supplemented with 10% fetal bovine serum (FBS, 10099158, Gibco, MA, USA) was used for culturing. Non-adherent cells were removed every other day, while adherent primary cells were subcultured once or twice until approximately 80% confluency. Cells in the high-fat and high-glucose (HFHS) group were treated with DMEM containing 30 mmol/L glucose (G8150, Solarbio, Beijing, China) and 0.3 mmol/L palmitic acid (N-16-A, Solarbio, Beijing, China). Control group cells were incubated in DMEM with a glucose concentration of 5.5 mmol/L. Bone marrow stromal cells were treated with LIR (100 nM) for 48 hours (20).

### 3.6. Plasmid Construction and Cell Transfection 

The SIRT1 interference expression vector shRNA was designed and synthesized by Gene Pharma (Shanghai, China). Cells were cultured in serum-free medium for 24 hours, after which Lipofectamine 2000 (11668030, Invitrogen, Carlsbad, CA, USA) and plasmids were mixed into the cells for transfection. Cells were transfected with sh-SIRT1 or NC, followed by RT-PCR analysis to assess silencing efficiency. The sequences for SIRT1 shRNA and NC are provided in Appendix 1 in the Supplementary File.

### 3.7. Cell Apoptosis Assay 

Transfected cells were collected in tubes for flow cytometric analysis. After centrifuging at 4°C at 1000 g for 5 minutes, the supernatant was discarded, and cells were resuspended in 195 µL of Annexin V-FITC binding buffer from the Annexin V-FITC Apoptosis Detection kit (CA1020, Solarbio, Beijing, China). To the cell suspension, 5 µL of Annexin V-FITC and 10 µL of PI were added, followed by a 20-minute incubation in the dark. Apoptosis was analyzed using a flow cytometer (FACScan, BD Biosciences).

Alizarin red staining was conducted using the Alizarin Red S Staining kit (A5533-25G, Sigma-Aldrich, Shanghai, China). According to the manufacturer’s instructions, after removing the culture medium, 1 × 10⁵ cells were rinsed twice with 1 mL PBS and fixed with 4% paraformaldehyde for 15 minutes. After removing the fixative, cells were rinsed three times with diH₂O. Following the removal of diH₂O, 1 mL of 2% Alizarin Red S Stain solution was added to each well and incubated for 30 minutes. The stain solution was then removed, and cells were rinsed with diH₂O three to five times. To prevent dehydration, 1 mL of distilled water was added to each well. The samples were observed under a light microscope (Olympus BX50 microscope, Tokyo, Japan).

### 3.8. RT-PCR Analysis 

Relative mRNA expression was measured by RT-PCR analysis. Total RNA was extracted from cell lysates using the PicoPure™ RNA Isolation Kit (KIT0204, Applied Biosystems, Foster City, CA, USA), and cDNA synthesis was carried out using the HiScript II 1st Strand cDNA Synthesis Kit (R211-01, Vazyme, Nanjing, China). Primer sequences are provided in Appendix 2 in the Supplementary File. Thermal cycling was performed on the ABI Prism 7500 (Applied Biosystems, Foster City, CA, USA) with 40 cycles at 95°C for 10 minutes, followed by 95°C for 15 seconds, 60°C for 15 seconds, and 72°C for 15 seconds. mRNA expression levels were normalized to GAPDH.

### 3.9. Detection of Oxidative Stress and Inflammatory Factors 

Levels of interleukin 6 (IL-6) (ERA31RB, Invitrogen, Carlsbad, CA, USA), TNF-α (ab236712, Abcam, Shanghai, China), IL-10 (ERA23RB, Invitrogen, Carlsbad, CA, USA), TGF-β (BMS623-3, Invitrogen, Carlsbad, CA, USA), malondialdehyde (MDA, ab287797, Abcam, Shanghai, China), and superoxide dismutase (SOD, EIASODC, Invitrogen, Carlsbad, CA, USA) were measured according to the respective ELISA kit instructions. The sensitivity and detection range of each ELISA kit are provided in Appendix 3 in the Supplementary File. Samples were incubated with the coating solution on ELISA plates for 2 hours, then sealed with 10% calf serum overnight at 4°C. Following washes, samples were treated with primary antibodies at 37°C for 2 hours, then with secondary antibodies for 1 hour at the same temperature. After the termination solution was added, optical density (OD) values were read at 450 nm using a spectrophotometer (UV-1780, Shimadzu, Japan).

### 3.10. Statistical Analysis 

Data are presented as mean ± standard deviation (SD). Statistical analysis was performed using GraphPad Prism 7.0 software (GraphPad Software, CA, USA). One-way analysis of variance (ANOVA), followed by post hoc tests, was used to assess statistical significance, with P-values below 0.05 considered statistically significant.

## 4. Results 

### 4.1. Liraglutide Regulates Streptozotocin-Induced Bone Metabolism Imbalance and Neutrophil Extracellular Traps Activation 

**Figure 1.**
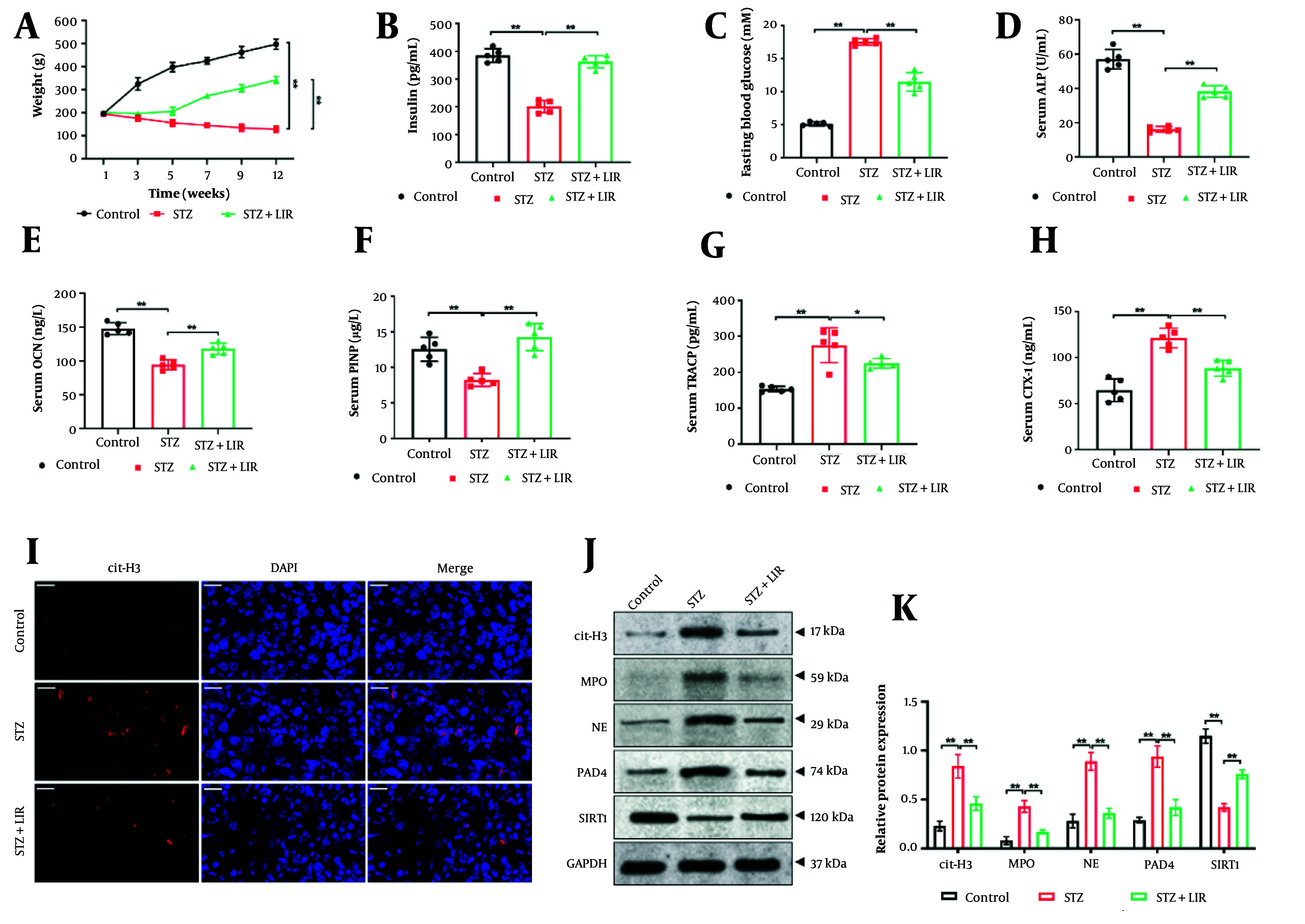
Liraglutide (LIR) regulated streptozotocin (STZ)-induced bone metabolism imbalance and activation of neutrophil extracellular traps (NETs). Weight changes in rats were monitored weekly (A); the levels of serum insulin (B); and fasting blood glucose (FBG) (C) were detected; the serum levels of alkaline phosphatase (ALP) (D); osteocalcin (OCN) (E); procollagen I N-terminal propeptide (PINP) (F); tartrate-resistant acid phosphatase (TRACP) (G); and CTX-1 (H) were assessed using the corresponding assay kits; expression of cit-H3 in rat femur tissues was determined via immunofluorescence staining, scale bar = 20 μm (I); western blotting assays detected the expression of Sirtuin-1 (SIRT1) and NETs formation markers [cit-H3, myeloperoxidase (MPO), NE, and PAD4] in femur tissues, with GAPDH serving as the reference gene (J); quantitative analysis of the protein expressions (K). * P < 0.05, ** P < 0.01. All data are presented as means ± SD.

The diabetic rat model was induced using STZ, and body weight was monitored for each group. Results (Figure 1A) indicated that the body weight of STZ model rats was significantly reduced compared to the control group, whereas LIR treatment resulted in a notable increase in body weight (P < 0.01). Blood insulin and FBG levels were measured across groups. The findings (Figure 1B, C) showed that insulin levels in the LIR treatment group were significantly elevated, while FBG levels were markedly reduced compared to the STZ group (P < 0.01).

To assess diabetes' effects on bone metabolism, we analyzed bone metabolism markers. Compared to the STZ group, bone formation markers (ALP, OCN, and PINP) were significantly increased in the LIR group (P < 0.01), whereas bone resorption markers (TRACP and CTX-1) were significantly decreased (Figure 1D-H). Additionally, to investigate whether NETs are involved in LIR's regulation of bone metabolism, immunofluorescence staining was performed to assess cit-H3 expression, a key marker of NETs formation. As shown in Figure 1I, STZ induction significantly increased cit-H3 expression, which was notably reduced by LIR treatment.

Given SIRT1's critical role in bone metabolism, we evaluated SIRT1 levels and NETs formation markers (cit-H3, MPO, NE, and PAD4) in femur tissues. The results (Figure 1J, K) indicated that LIR reduced STZ-induced NETs formation, with decreases in cit-H3 by 36.90% - 53.57%, MPO by 55.81% - 65.12%, NE by 53.95% - 65.17%, and PAD4 by 46.81% - 63.83%, alongside a significant increase in SIRT1 expression (70.71% - 91.19%) in femur tissue. These findings suggest that LIR effectively mitigates diabetes-induced abnormalities in bone metabolism, potentially through the regulation of NETs and SIRT1.

### 4.2. Knockdown of Sirtuin-1 Reverses Apoptosis Inhibition and Osteogenic Capability Induced by Liraglutide in Bone Marrow Mesenchymal Stem Cells 

**Figure 2.**
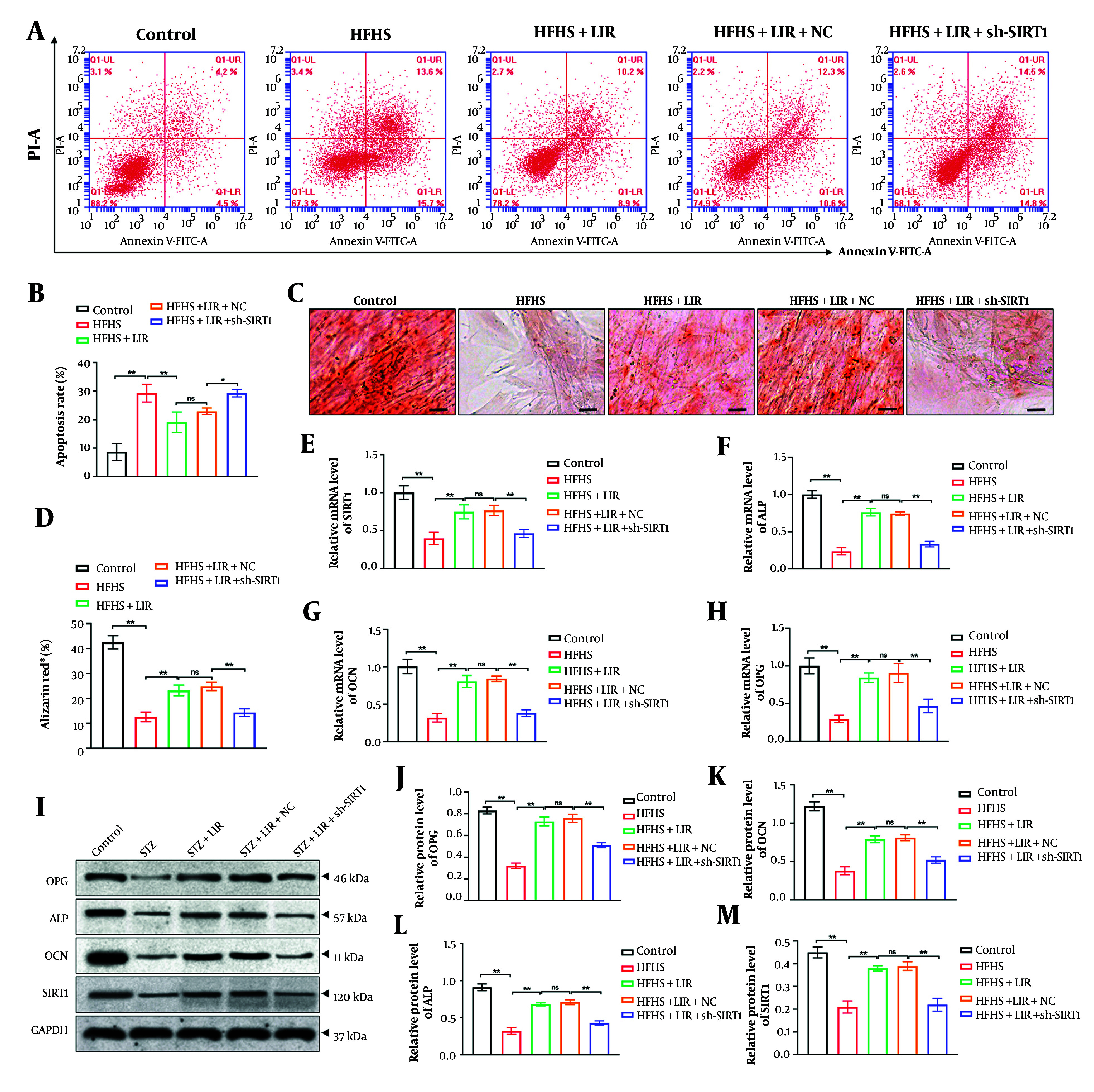
Knockdown of sirtuin-1 (SIRT1) reversed apoptosis inhibition and osteogenic capability induced by liraglutide (LIR) in bone marrow mesenchymal stem cells (BMSCs). Apoptosis assays were performed using flow cytometric analysis (A, B); the calcium deposition of BMSCs was evaluated by Alizarin Red staining, scale bar = 20 μm (C, D); the mRNA expressions of SIRT1 (E); alkaline phosphatase (ALP) (F); osteocalcin (OCN) (G; and OPG (H) were examined through RT-PCR analysis; western blotting assays detected the expression of SIRT1, ALP, OCN, and OPG in BMSCs, with GAPDH serving as the reference gene (I); quantitative analysis of the protein expressions (J-M). * P < 0.05, ** P < 0.01. ns = non-significant. All data are presented as means ± SD.

Next, we constructed SIRT1 shRNA (sh-SIRT1) and transfected it into BMSCs for 48 hours. The knockdown efficiency was evaluated via RT-PCR analysis, and the results (Appendix 4 in the Supplementary File) showed that sh-SIRT1-2 exhibited the best efficiency, which was selected for the subsequent experiments. We subjected BMSCs to HFHS treatment and observed cell apoptosis after LIR treatment, both with and without sh-SIRT1 transfection. As shown in Figure 2A and B, LIR significantly suppressed cell apoptosis induced by HFHS treatment (P < 0.01). However, sh-SIRT1 transfection effectively reversed the impact of LIR and increased BMSC apoptosis (P < 0.05).

Additionally, the osteogenic differentiation of BMSCs was assessed through Alizarin Red staining. The findings (Figure 2C, D) revealed that, compared to the control group, HFHS induction led to fewer calcium deposits, which were effectively reversed by LIR treatment (P < 0.01). However, the knockdown of SIRT1 significantly reduced the effects of LIR and decreased calcium deposition (P < 0.01).

Moreover, we investigated the expression of SIRT1 and bone metabolism markers (OPG, ALP, and OCN) using RT-PCR and Western blot assays, respectively. The results indicated that transfection of sh-SIRT1 remarkably reversed LIR-enhanced mRNA expression (Figure 2E-H) and protein levels (Figure 2I-M) of SIRT1, OPG, ALP, and OCN (P < 0.01). This supports the hypothesis that SIRT1 plays a crucial role in mediating the effects of LIR on cell apoptosis and bone metabolism. The results underscore the importance of SIRT1 in the therapeutic action of LIR in counteracting HFHS-induced cellular and metabolic abnormalities.

### 4.3. Knockdown of Sirtuin-1 Regulates Liraglutide-Inhibited Oxidative Stress and Inflammation In Vivo 

**Figure 3.**
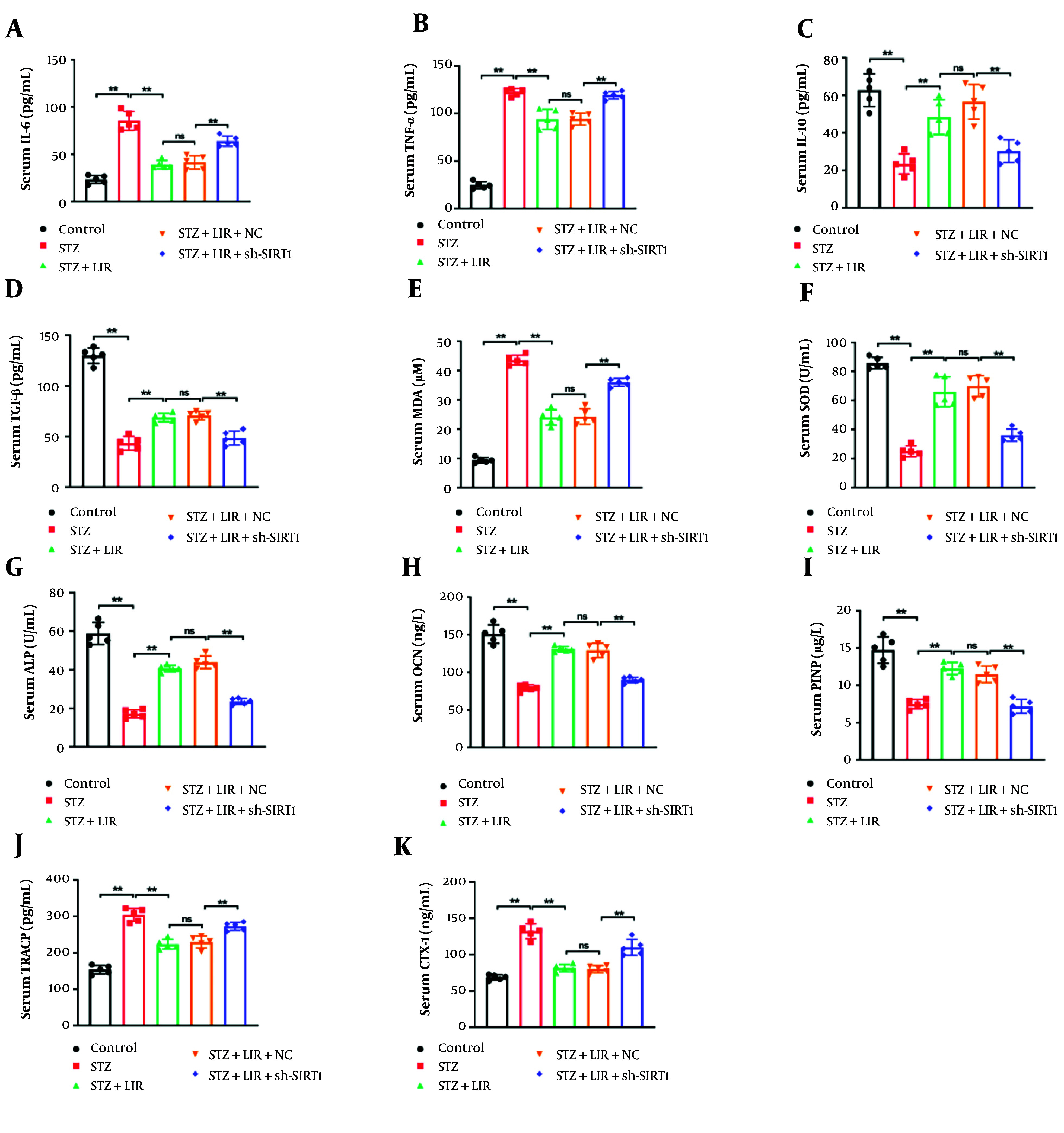
Knockdown of sirtuin-1 (SIRT1) regulated liraglutide (LIR)-inhibited oxidative stress and inflammation in vivo. The serum pro-inflammatory factors interleukin 6 (IL-6) (A); and TNF-α (B; as well as the anti-inflammatory factors IL-10 (C); and TGF-β (D); were detected using the corresponding assay kits. The levels of malondialdehyde (MDA) (E); and superoxide dismutase (SOD) (F) were assessed through ELISA kits; the serum levels of alkaline phosphatase (ALP) (G); OCN (H); procollagen I N-terminal propeptide (PINP) (I); tartrate-resistant acid phosphatase (TRACP) (J); and CTX-1 (K) were assessed using the corresponding assay kits. * P < 0.05, ** P < 0.01. ns = non-significant. All data are presented as means ± SD.

We further conducted in vivo experiments to confirm whether LIR regulates bone metabolism by targeting SIRT1. Firstly, we assessed the inflammation and oxidative stress in rat serum samples. The findings showed that LIR decreased the pro-inflammatory factors IL-6 (Figure 3A), (P < 0.01) and TNF-α (Figure 3B), (P < 0.01), while promoting the anti-inflammatory factors IL-10 (Figure 3C), (P < 0.01) and TGF-β (Figure 3D), (P < 0.01). The knockdown of SIRT1 significantly reversed the anti-inflammatory effects of LIR. Additionally, the reduced levels of MDA (Figure 3E) and the enhanced levels of SOD (Figure 3F) caused by LIR were notably reversed by sh-SIRT1 transfection (P < 0.01). 

Furthermore, compared with the LIR group, the levels of ALP (Figure 3G) (P < 0.01), OCN (Figure 3H), (P < 0.01), and PINP (Figure 3I) (P < 0.01) significantly decreased after the knockdown of SIRT1, while the levels of TRACP (Figure 3J), (P < 0.01) and CTX-1 (Figure 3K), (P < 0.01) significantly increased. These results revealed that SIRT1 is crucial for the anti-inflammatory and bone-regulatory actions of LIR. This is consistent with the hypothesis that LIR exerts its therapeutic effects on bone metabolism through modulation of SIRT1.

### 4.4. Down-Regulation of Sirtuin-1 Enhanced the Liraglutide-Inhibited Neutrophil Extracellular Traps Level In Vivo 

**Figure 4.**
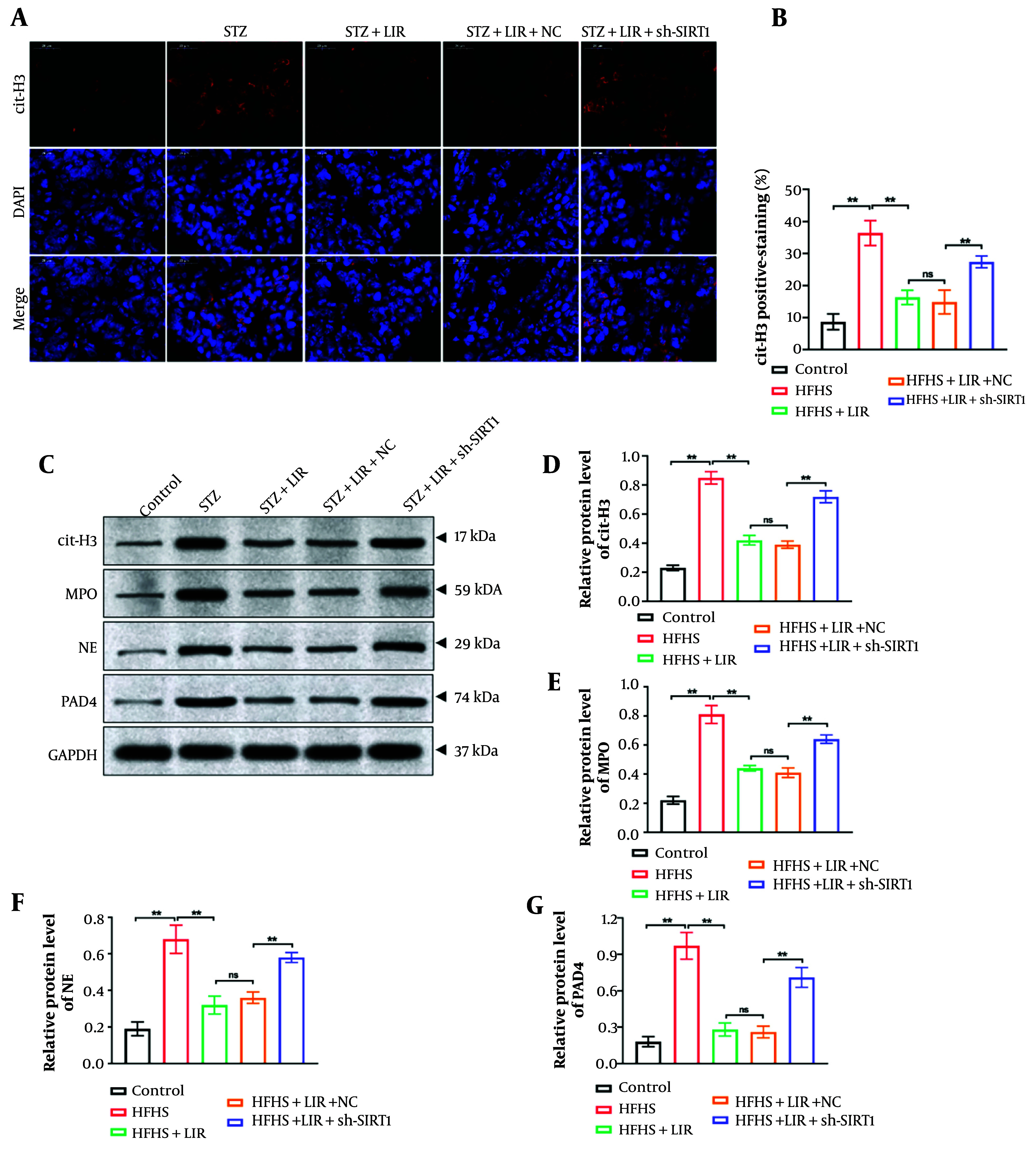
Down-regulation of sirtuin-1 (SIRT1) enhanced the liraglutide (LIR)-inhibited neutrophil extracellular traps (NETs) level in vivo. Expression of cit-H3 in rat femur tissues was determined via immunofluorescence staining, scale bar = 20 μm (A); quantification of cit-H3 fluorescence intensity was analyzed (B); western blotting assays detected the expression of cit-H3, myeloperoxidase (MPO), NE, and PAD4 in femur tissues, with GAPDH serving as the reference gene (C); quantitative analysis of the protein expressions (D, G). * P < 0.05, ** P < 0.01. All data are presented as means ± SD.

Immunofluorescence staining was performed to determine the level of cit-H3 in femur tissues. As shown in Figure 4A and B, compared to the LIR group, the knockdown of SIRT1 significantly promoted the expression of cit-H3, which was inhibited by LIR (P < 0.01). Moreover, a Western blot assay was conducted to explore the expressions of NETs formation markers (cit-H3, MPO, NE, and PAD4) in rat femur samples. The findings (Figure 4C-G) demonstrated that sh-SIRT1 transfection remarkably reversed LIR-induced NETs formation (P < 0.01). These findings support the hypothesis that SIRT1 is a key regulator of NETs formation and that LIR’s effects on NETs are mediated through SIRT1 modulation.

## 5. Discussion 

Diabetes frequently correlates with compromised bone health and disruptions in calcium and phosphorus levels, leading to conditions such as secondary osteopenia, osteoporosis, and other bone diseases commonly seen in individuals with diabetes (21). Diabetes hinders the process of bone formation, increases the likelihood of fractures, and slows the healing of fractures (22). Patients with DM often exhibit an imbalance in bone metabolism and are predisposed to osteoporosis in their jaw bones (23). This study aimed to investigate whether LIR regulates bone metabolism through the modulation of SIRT1 and to understand its effects on inflammation, oxidative stress, and NETs formation. We hypothesized that LIR regulates bone metabolism through SIRT1 by enhancing bone formation and reducing bone resorption. Additionally, we posited that SIRT1 plays a crucial role in mediating LIR’s inhibition of NETs formation, with SIRT1 knockdown reversing this effect. Our research demonstrated that LIR treatment successfully increased levels of bone formation markers and decreased the expression of bone resorption markers induced by STZ, both in vitro and in vivo. Mechanistically, we showed that SIRT1-mediated NETs formation plays a crucial role in LIR-regulated bone metabolism in diabetic rats. 

Neutrophils are instrumental in attracting mononuclear leukocytes to initiate an immune response at the site of injury (23). They significantly impact inflammation by releasing cytokines and chemokines, degranulating to release MPO and NE, and forming NETs composed of large DNA networks (24). An ELF-PEMF at 16 Hz has been found to be a non-invasive treatment that aids in bone healing without inducing ROS and Ca²⁺ influx in neutrophils (25). ROS and Ca²⁺ influx can trigger neutrophils to produce excessive amounts of NETs, which can adversely affect the healing process (25). Patients with rheumatoid arthritis exhibit elevated levels of NETs in the synovial fluid compared to those with osteoarthritis, which is associated with higher levels of RANKL/OPG (26). Alleviating bone loss in arthritic mice has been achieved by inhibiting NETs through DNase treatment or deletion of Padi4 (26). In rheumatoid arthritis, osteoclast formation is induced by NETs through toll-like receptor 4 signaling and NET-associated proteins such as histones and NE (27). Neutrophil extracellular traps induced by monosodium urate crystals can inhibit osteoblast viability and enhance osteoclast activity, further inducing an imbalance between RANKL and OPG (28).

Research has reported that LIR regulates the progression of various diseases through the SIRT1 pathway. Liraglutide reduces body weight gain, excessive lipid accumulation, and improves muscle atrophy induced by a high-fat diet through the SIRT1 pathway (29). Additionally, LIR has the capability to restore impaired glucose tolerance and insulin resistance (29). It mitigated cellular senescence in human retinal endothelial cells (HRECs) stimulated by high glucose (HG) through the activation of SIRT1 (30). Liraglutide also reduces the HG-induced upregulation of vascular endothelial growth factor-A (VEGF-A) and IL-6 (30). In our study, LIR influenced bone metabolism through a SIRT1-mediated pathway by enhancing bone formation, reducing bone resorption, and improving the inflammatory and oxidative stress environment. It may also affect NETs formation, with SIRT1 playing a critical role in modulating this process. Enhanced SIRT1 activity due to LIR treatment could reduce NETs formation and associated inflammation, contributing to better bone health.

Due to funding constraints, we did not explore the further molecular mechanisms of NETs on bone metabolism in DM rats in this study. Significant work will be performed to obtain an in-depth understanding of this topic, including the impacts of other gene regulatory networks on NETs. Further studies also need to enlarge the sample size to support the clinical application of NETs inhibitors on DM-induced bone metabolism imbalance.

Improving bone formation and reducing markers of bone resorption can significantly benefit diabetics by lowering the risk of fractures and enhancing bone healing. Increased bone formation boosts bone density and quality, making bones stronger and less prone to fractures, while reduced bone resorption preserves bone mass and stability. Together, these effects lead to a lower incidence of fractures, faster and more effective bone repair, improved quality of life, and decreased healthcare costs related to bone injuries. Thus, targeting these pathways can substantially enhance bone health and recovery outcomes for individuals with diabetes. To summarize, the present research reveals that LIR significantly promotes the correction of DM-induced bone metabolism imbalance through SIRT1-mediated NETs generation. Hence, our findings may elucidate a new regulatory mechanism of LIR and SIRT1 in DM. 

ijpr-23-1-148139-s001.pdf

## Data Availability

The data utilized and/or analyzed throughout this research are available from the corresponding author upon reasonable request.
