# Supplementary material for: The Glucagon-Like Peptide-1 (GLP-1) Receptor Agonist Liraglutide Regulates Sirtuin-1-Mediated Neutrophil Extracellular Traps to Improve Diabetes-Induced Bone Metabolism Imbalance
Source: Iran J Pharm Res. 2024 Nov 13;23(1):e148139. doi: 10.5812/ijpr-148139 (PMC11892751; doi:10.5812/ijpr-148139)
Supplement: ijpr-23-1-148139-s001.pdf [file ijpr-23-1-148139-s001.pdf]

Appendix 1. The SIRT1 shRNA sequences.

**shRNAs**	
SIRT1 shRNA-1	5′-GGCAGTTAATGAAGCTATA-3′
SIRT1 shRNA-2	5′-GAACAAAGTTGACGATTTA-3′
SIRT1 shRNA-3	5′-GGTTTAGTATTTATTACAA-3′
NC	5′-TTCTCCGAACGTGTCACGT-3′

Appendix 2. Primers used for RT-PCR in this study.

**Primer sequence**		
SIRT1	Forward	5′-CAAAATCCAGCAACTCAGCA-3′
	Reverse	5′-GATCCTTTGGATTCCTGCAA-3′
ALP	Forward	5′- ACTGGTACTCAGACAACGAGAT-3′
	Reverse	5′- ACGTCAATGTCCCTGATGTTATG-3′
OCN	Forward	5′- GGCGCTACCTGTATCAATGG-3′
	Reverse	5′- GTGGTCAGCCAACTCGTCA-3′
OPG	Forward	5′- CAAAGTAAACGCAGAGAGTGTAGA-3′
	Reverse	5′- GAAGGTGAGGTTAGCATGTCC-3′
GAPDH	Forward	5′- AGACAGCCGCATCTTCTTGT-3′
	Reverse	5′- CTTGCCGTGGGTAGAGTCAT-3′

Appendix 3. ELISA detection sensitivity and detection range.

Biomarker	*Intra-assay* CV (%)	*Inter-assay* CV (%)	Detection sensitivity	Detection range
IL-6	<10%	<12%	20 pg/mL	20-10,000 pg/mL
TNF-α	3.9%	5.1%	1.04 pg/mL	18.75 - 1200 pg/mL
IL-10	<10%	<12%	10 pg/mL	10-6,000 pg/mL
TGF-β	6.8%	6.8%	7.8 pg/mL	31.25-2,000 pg/mL
MDA	< 8%	< 10%	4.688 µM	7.813µM - 500µM
SOD	4.6%	10.5%	0.044 U/mL	0–4 U/mL

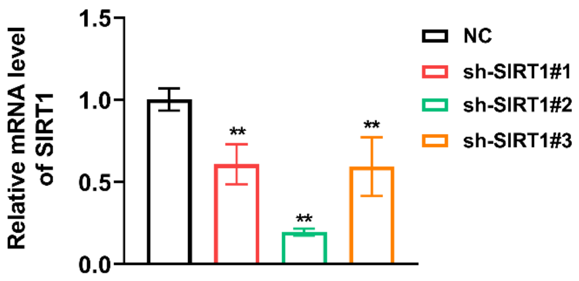

Appendix 4. The knockdown efficiency was evaluated through RT-PCR analysis. And sh-SIRT1-2 had the best efficiency, which was chosen for the next experiments.
